# Prognostic Significance of the Stage at Which an MRD-Negative Status Is Achieved for Patients With Multiple Myeloma Who Received ASCT

**DOI:** 10.3389/fonc.2022.776920

**Published:** 2022-05-18

**Authors:** Qian Sun, Xiaozhe Li, Jingli Gu, Beihui Huang, Junru Liu, Meilan Chen, Juan Li

**Affiliations:** Department of Hematology, The First Affiliated Hospital of Sun Yat-sen University, Guangzhou, China

**Keywords:** autologous stem cell transplantation, minimal residual disease, multiple myeloma, overall survival, time to progression

## Abstract

**Objective:**

To explore the prognostic significance of the stage at which a minimal residual disease (MRD)-negative status is achieved for patients with newly diagnosed multiple myeloma (NDMM) who received autologous hematopoietic stem cell transplantation (ASCT).

**Cases and Methods:**

A retrospective analysis of 186 NDMM patients who received “induction therapy-ASCT-maintenance therapy” in our center and achieved an MRD-negative status was performed. Patients were divided into three groups, A (induction therapy), B (3 months after ASCT), and C (maintenance therapy), according to the stage at which an MRD-negative status was achieved.

**Results:**

The median time to progression (TTP) of 186 patients was not reached; the median overall survival (OS) was 113.8 months. The median TTP of the patients in three groups was not reached (P=0.013), and the median OS of the patients in three groups was not reached, not reached, and 71.2 months, respectively (P=0.026). Among patients with standard-risk cytogenetics, the median TTP of those in all three groups was not reached (P=0.121), and the median OS of the patients in three groups was not reached, not reached, and 99.6 months, respectively (P=0.091). Among patients with high-risk cytogenetics, the median TTP of those in three groups was not reached, 53.9 months, and 35.8 months (P=0.060), and the median OS was not reached, 71.2 months, and 60.2 months, respectively (P=0.625). Among patients with R-ISS stage I-II, the median TTP of those in three groups was not reached (P=0.174), and the median OS of the patients in three groups was not reached, not reached, and 99.6 months, respectively (P=0.186). Among the 29 patients with R-ISS stage III, the median TTP of those in the 3 groups were unreached, unreached, and 35.1 months (P<0.001), and the median OS was unreached, unreached, and 48.5 months, respectively (P=0.020). In all enrolled patients, the stage of reaching MRD-negative was an independent prognostic factor for TTP, rather than a prognostic factor for OS. The stage of reaching MRD-negative in patients with R-ISS III was an independent prognostic factor for OS.

**Conclusion:**

For the same patients who are MRD-negative, the prognoses of those who achieve an MRD-negative status at different groups are different. The stage at which an MRD-negative status is achieved can predict the prognosis of patients with R-ISS stage III.

## Introduction

Multiple myeloma (MM) is a hematologic malignancy characterized by the accumulation of malignant plasma cells, renal insufficiency, hypercalcemia, anemia, and bone destruction. In the past two decades, the availability of new drugs and autologous hematopoietic stem cell transplantation (ASCT) for MM has led to significantly improved long-term outcomes (1). “Induction therapy-autologous hematopoietic stem cell transplantation-maintenance therapy” has become the preferred treatment option for MM patients eligible for ASCT (1, 2).

Minimal residual disease (MRD) assessment by multiparameter flow cytometry (MFC) on bone marrow is a sensitive strategy to accurately measure the response (3–6). At the end of each treatment stage, traditional efficacy and MRD need to be evaluated (1). Patients who are MRD-negative have a better prognosis (2, 3, 7). Regardless of the stage at which an MRD-negative status is achieved, the lower the MRD level detected with a highly sensitive technique is, the longer the progression-free survival (PFS) and overall survival (OS) periods (4, 8). The pretransplantation MRD level was an independent prognostic factor for event-free survival (EFS) and OS (9). MRD status by MFC at day 100 or 365 after ASCT was the most important independent prognostic factor for PFS and OS (3, 10). These findings demonstrate the clinical importance of MRD evaluation, and the MRD-negative status has become the goal of current treatment, and it has also become the endpoint of many clinical trials (11, 12).

However, previous studies were mostly based on the prognostic significance of MRD results at a single time point. It is believed that regardless of which stage the MRD is negative, the prognosis is better than that of the positive ones (4, 8). A few studies combined multiple MRD results at different time points to predict the prognosis of MM, and as our previous study found, patients with a sustained negative MRD status lasting for at least 24 months had a better prognosis (13). For MRD-negative patients, it is not clear whether there is a difference in clinical features and prognosis after reaching an MRD-negative status at different stages, especially for high-risk cytogenetics patients. To clarify this issue, we conducted a retrospective clinical study.

## Materials and Methods

### Research and Design

Patients with newly diagnosed multiple myeloma (NDMM) who received “induction therapy-ASCT-maintenance therapy” at the First Affiliated Hospital of Sun Yat-sen University between January 2007 and January 2021 were evaluated for inclusion in our retrospective study. Patients were divided into three groups, A (induction therapy), B (3 months after ASCT), and C (maintenance therapy), according to the stage at which an MRD-negative status was achieved. The clinical characteristics and prognoses of patients in the three stages were compared. All patients met the International Myeloma Working Group (IMWG) diagnostic criteria, and a total of 186 patients were analyzed (2). The inclusion criteria were as follows: 1) NDMM with complete clinical data; 2) received induction therapy-ASCT maintenance treatment, and 3) curative effect was evaluated and MRD was negative. The exclusion criteria were as follows: 1) past tumor history or diagnosis of combined tumors; 2) severe complications that may affect treatment or survival; 3) received double ASCT; 4) all MRD detection results were positive, and 5) discontinued treatment at any stage. It should be pointed out that for patients with rapid progression after treatment, if they achieved MRD-negative before, they were included in the analysis, otherwise they were excluded. In this study, those with 17p-, t(4;14) or t(14;16) were allocated to the high-risk cytogenetics group, and those without the abovementioned cytogenetic changes were allocated to the standard-risk cytogenetics group (1).

### Sample Size Estimation

In this study, the patients were divided into three groups. The prognosis of the three groups of patients in different stages was compared, and the observed events were progression and death. The Kaplan-Meier survival curve was used to analyze the prognostic characteristics and the Log-Rank test was used. The study design conformed to the 3-rank vs. outcome event chi-square test model. The Df is set to 2, 1-β is set to 0.8, α is set to 0.05, w is set to 0.25 (medium effect size), and the sample size n=155 is calculated. When the test reaches the expected statistical significance, the ideal statistical test power can be achieved when the number of valid study cases is more than 155 cases.

### Treatment Plan

Induction therapy comprised a regimen containing bortezomib or lenalidomide. Some patients were treated with a regimen containing bortezomib and lenalidomide at the same time, with a median induction of 4 (1–12) courses. The mobilizing regimen was cyclophosphamide (CTX 3 g/m^2^, iv drip d1) + granulocyte colony-stimulating factor (G-CSF, 300 μg, ih QD from d2) or G-CSF alone (G-CSF, 300 μg, ih QD d1–5). The pretreatment regimen was high-dose melphalan (melphalan 200 mg/m^2^, reduction in renal insufficiency) or CVB (CTX 50 mg/kg, iv drip, QD -3 d~-2 d; etoposide 10 mg/kg, iv drip, QD, -5 d~-4 d; busulfan 0.8 mg/kg, iv, q6h, -8 d~-6 d). The following numbers of collected cells were required: MNCs>2.0×10^8^/kg and CD34+ cells>2.0×10^6^/kg. Maintenance treatment was started as soon as the blood cells recovered after transplantation. Proteasome inhibitors, immunomodulators, interferon, glucocorticoids, and Daratumumab were applied individually or in combination for maintenance treatment.

### Efficacy Evaluation

The IMWG 2016 efficacy standard was used to evaluate efficacy (2). The abovementioned efficacy evaluation was performed after induction chemotherapy, every 3 months in the first year after ASCT, and every six months after 1 year. At the same time, flow cytometry was used for MRD detection in the bone marrow. The antibody marker of flow cytometry MRD was CD38/CD45/CD19/CD20/CD56/CD54/CD138/cκ/cλ. The number of cells detected was 1×10^6^, the detection of ≥20 abnormal phenotype plasma cells was considered positive, and the limit of quantification (LOD) was 2×10^-5^ to 10^-5^.

### Follow-Up

Follow-up was conducted by telephone. All patients were followed up until January 31, 2021.

### Statistical Analysis

Sample size was estimated by G*Power version 3.0 (Heinrich-Heine-Universität Düsseldorf, Düsseldorf, German). Statistical analyses were carried out using SPSS 26.0 software (SPSS, Chicago, IL, US) and GraphPad Prism (version 9.0; GraphPad Software, La Jolla, California). Baseline characteristics were evaluated using descriptive statistical analysis: frequency distributions (n, %) are presented for categorical variables and compared using the chi-squared test. The median (range) is presented for continuous variables and compared using a nonparametric test, only age was a continuous variable in our study. Time to progression (TTP) was calculated from the start of treatment to disease progression or the last follow-up, and overall survival (OS) was calculated from the start of treatment until death or the last follow-up. The Kaplan-Meier method was used to analyze the survival of patients who achieved an MRD-negative status at different stages. P<0.05 indicated that the difference was statistically significant.

## Results

### Characteristics of the Included Patients

**Figure 1:**
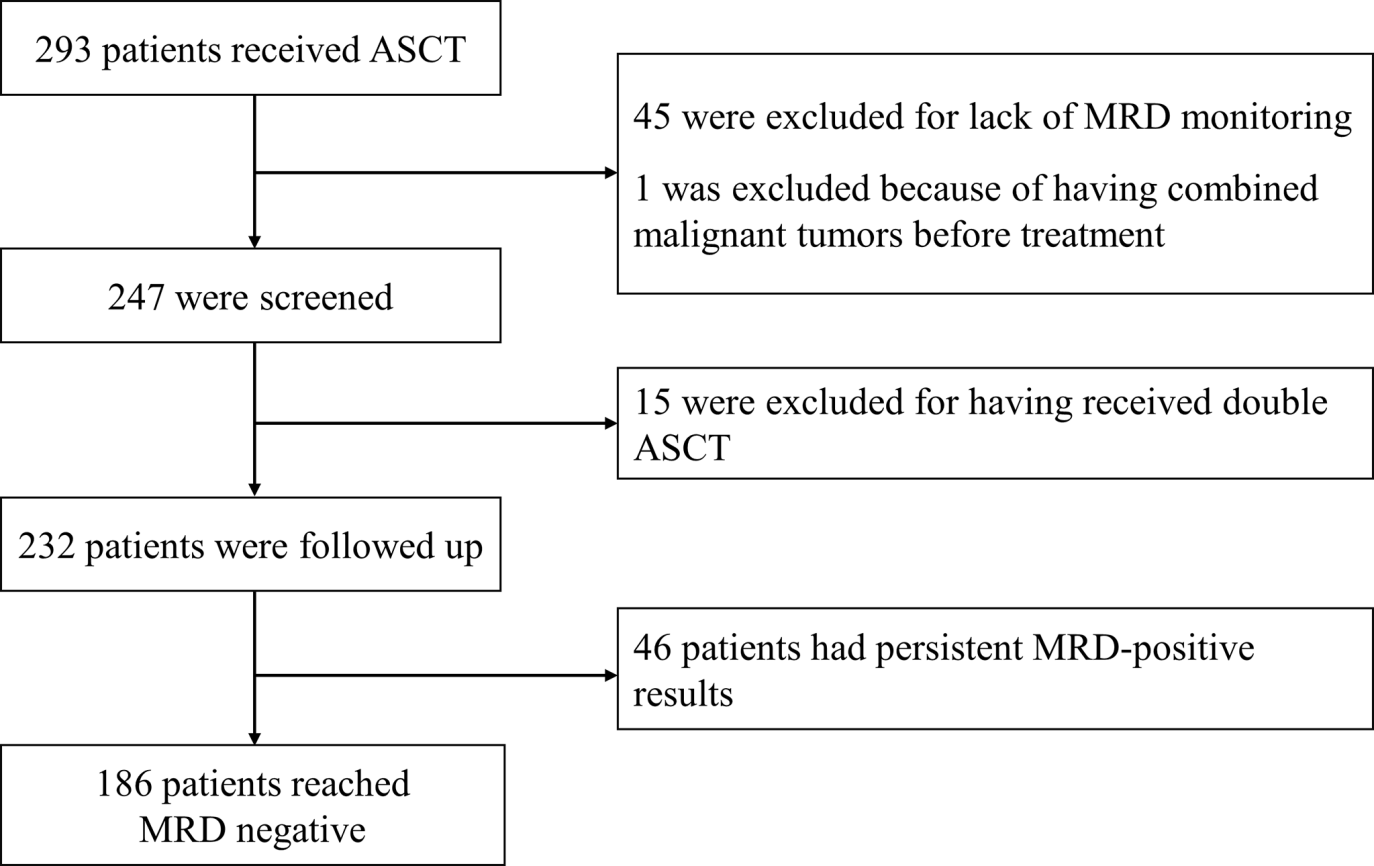
Patients included in this study.

This study reviewed 293 patients, 45 of whom were excluded due to a lack of MRD monitoring, 1 of whom was excluded due to having combined malignant tumors, 15 of whom received double ASCT, and 46 of whom had persistent MRD-positive results. Ultimately, 186 patients achieved an MRD-negative status, as shown in **Figure 1**.

The basic data of the enrolled patients are shown in **Table 1**. A comparison of the baseline information of patients in the three groups (A-C) is shown in **Table 2**. There was no significant difference in basic data between the groups (P>0.05).

**Table 1:** Baseline information of the 186 NDMM patients at disease onset.

Characteristic	n or Median	Ratio (%)/Range
Age (years); Median (Range)	54	26–69
Sex (n)		
Male	114	61.3
Female	72	38.7
Monoclonal protein type (n)		
IgG	88	47.3
IgA	49	26.3
IgD	6	3.2
Light chain disease	43	23.1
R-ISS stage (n)		
I-II	157	84.4
III	29	15.6
Hemoglobin (g/L)	96	44–173
Lactate dehydrogenase (U/L)	154	51–517
Bone marrow plasmacytosis (%)	21.0	0.5–85.0
Renal function stage (n)		
A	153	82.3
B	33	17.7
Cytogenetic abnormality (n)		
t(14;16)	6/162	3.7
t(4;14)	26/162	16.0
t(11;14)	16/162	9.9
del(13q)	54/163	33.1
del(17p)	12/152	7.9
1q21 gain	54/148	36.5
Amyloidosis (n)	11	5.9
High-risk cytogenetics*(n)	38/162	23.5

^*^High-risk cytogenetics reference for t(4;14), t(14;16) and 17p-.

**Table 2:** Comparison of basic clinical information from three groups of patients.

Characteristic	Group A (n=73)	Group B (n=42)	Group C (n=71)	P
Age (years)	53.0	56.0	54.0	0.611
Hb (g/L)	97.0	91.0	98.0	0.614
β2-MG (μg/L)	3256.0	3030.7	3343.4	0.949
ALB (g/L)	34.7	36.0	34.7	0.398
CREA (μmol/L)	85.0	90.0	90.0	0.364
LDH (U/L)	167.0	168.0	153.0	0.748
Bone marrow plasmacytosis (%)	18.3	25.0	23.5	0.338
Conditioning regimens (n)				0.726
CVB	40	21	41	
HD-Mel	33	21	30	
CD34+ cells (×10^6^/kg)	2.9	3.5	3.0	0.529
R-ISS (n)				0.892
I-II	61	35	61	
III	12	7	10	
Cytogenetics abnormality (n)				0.627
Standard risk	54	24	46	
High risk	16	10	12	
Type of myeloma (n)				0.870
IgG	38	20	30	
IgA	16	12	21	
IgD	2	2	2	
Light chain only	17	8	18	

### Treatment Process and Efficacy Evaluation

A total of 178 patients received a bortezomib-containing regimen for induction therapy, 19 patients were treated with a lenalidomide-containing regimen for induction therapy, and 11 patients were treated with a bortezomib- and lenalidomide-containing regimen for induction therapy. Eighty patients (43.0%) achieved complete response (CR) after induction therapy, 81 (43.5%) achieved very good partial response (VGPR), 21 (11.3%) achieved partial response (PR), and 4 (2.2%) did not achieve PR. Seventy-three patients (39.2%) achieved an MRD-negative status after induction therapy, and another 113 patients (60.8%) remained MRD positive at this stage. A total of 180 patients received high-dose CTX+G-CSF for peripheral blood stem cell mobilization, and 15 patients failed after the first mobilization; of these patients, peripheral blood stem cells were successfully collected in 5 after receiving the same protocol again, peripheral blood + bone marrow stem cells were collected in 4 after receiving the same protocol again, and peripheral blood + bone marrow stem cells were collected after mobilization with G-CSF monotherapy in 6. Another 6 patients were mobilized with G-CSF (300 µg/d, d1–5) to collect bone marrow stem cells. 102 patients received CVB pretreatment; 69 patients received high-dose melphalan pretreatment, and 15 patients received a reduced-dose Mel regimen due to renal insufficiency[100mg/m^2^ (n=3), 120mg/m^2^ (n=1), 140mg/m^2^ (n=10), 150mg/m^2^ (n=1)]. The median count of CD34^+^ cells was 3.06 (0.13–17.8)×10^6^/kg, the median reconstruction time of granulocytes was 10 d, and the median reconstruction time of megakaryocytes was 11 d. Maintenance therapy with bortezomib + thalidomide + dexamethasone after ASCT (n=1); Bortezomib+Dexamethasone (n=11); Daratumumab (n=1); Thalidomide (n=121); Interferon (n=31); lenalidomide (n=17); Ixazomib (n=4). Forty-two patients (22.6%) achieved an MRD-negative status after transplantation. The best curative effect after maintenance treatment was CR in 163 patients (87.6%), VGPR in 22 patients (11.8%), and PR in 1 patient (0.5%). Seventy-one patients (38.2%) achieved an MRD-negative status after maintenance treatment.

### Survival in All Patients

**Figure 2:**
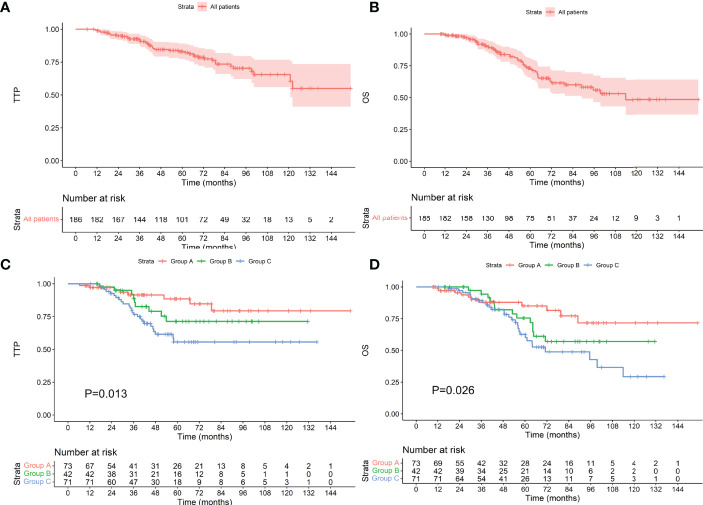
TTP **(A)** and OS **(B)** for all patients included. TTP **(C)** and OS **(D)** for patients who achieved an MRD-negative status at different stages. The Group A, Group B, and Group C represent achieving an MRD-negative status after induction therapy, ASCT, and maintenance therapy, respectively (the same below).

The median follow-up time was 67.6 (9.3–154.9) months. The median TTP of 186 patients was not reached, and the median OS was 113.0 months. Of the 73 patients who achieved an MRD-negative status after induction, 8 progressed during the follow-up period, 11 died, and neither TTP nor OS was reached; of the 42 patients who achieved an MRD-negative status after ASCT, 9 progressed and 13 died. The TTP and OS were not reached. Among the 71 patients who achieved an MRD-negative status after maintenance, 25 progressed, and 28 died. The TTP was not reached, and OS was 71.2 months. As shown in **Figure 2**, there were differences in TTP and OS between the groups (P values were 0.013 and 0.026, respectively).

### Survival in Patients With Different Cytogenetics

**Figure 3:**
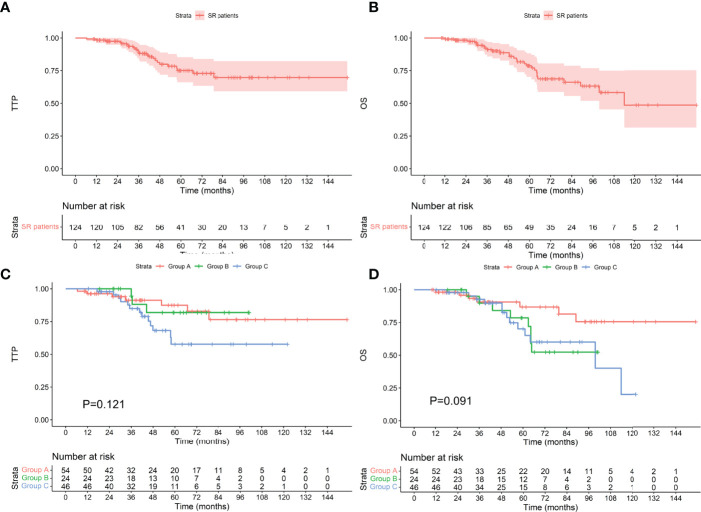
TTP **(A)** and OS **(B)** for patients with standard-risk cytogenetics and TTP **(C)** and OS **(D)** for patients who achieved an MRD-negative status at different stages. SR, standard-risk cytogenetics.

**Figure 4:**
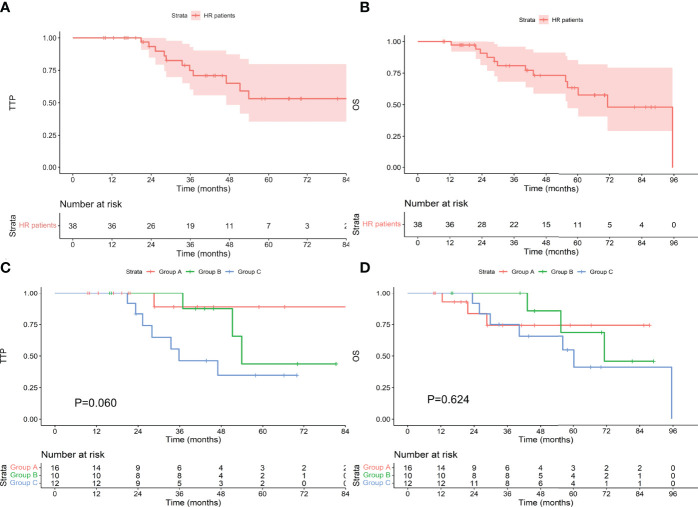
TTP **(A)** and OS **(B)** for patients with high-risk cytogenetics and TTP **(C)** and OS **(D)** for patients who achieved an MRD-negative status at different stages. HR, high-risk cytogenetics.

Among the 124 patients with standard-risk cytogenetics, the median TTP was not reached, and the median OS was 113.8 months. Among the 54 patients who achieved an MRD-negative status after induction, 7 progressed during the follow-up period, and 7 died. The median TTP and median OS were not reached. Among the 24 patients who achieved an MRD-negative status after ASCT, 3 progressed, and 8 died. The median TTP and median OS were not reached. Among the 46 patients who achieved an MRD-negative status after maintenance, 13 progressed, and 13 died. The median TTP was not reached, and the median OS was 99.6 months. There was no difference in TTP or OS between the groups (as shown in **Figure 3**, P values were 0.121 and 0.091, respectively). Among the 38 patients with high-risk cytogenetics, among the 16 who achieved an MRD-negative statusafter induction, 1 progressed during the follow-up period, and 3 died. The median TTP and median OS were not reached. Among the 10 patients who achieved an MRD-negative status after ASCT, 3 progressed, and 3 died. The median TTP was 53.9 months, and the median OS was 71.2 months. Among the 12 patients who achieved an MRD-negative status after maintenance, 7 progressed, and 7 died. The median TTP was 35.8 months, and the median OS was 60.2 months. There was no difference in TTP or OS between the groups (as shown in **Figure 4**, P values were 0.060 and 0.624, respectively).

### Survival in Patients With t(4;14), t(14;16), and 17p-

**Figure 5:**
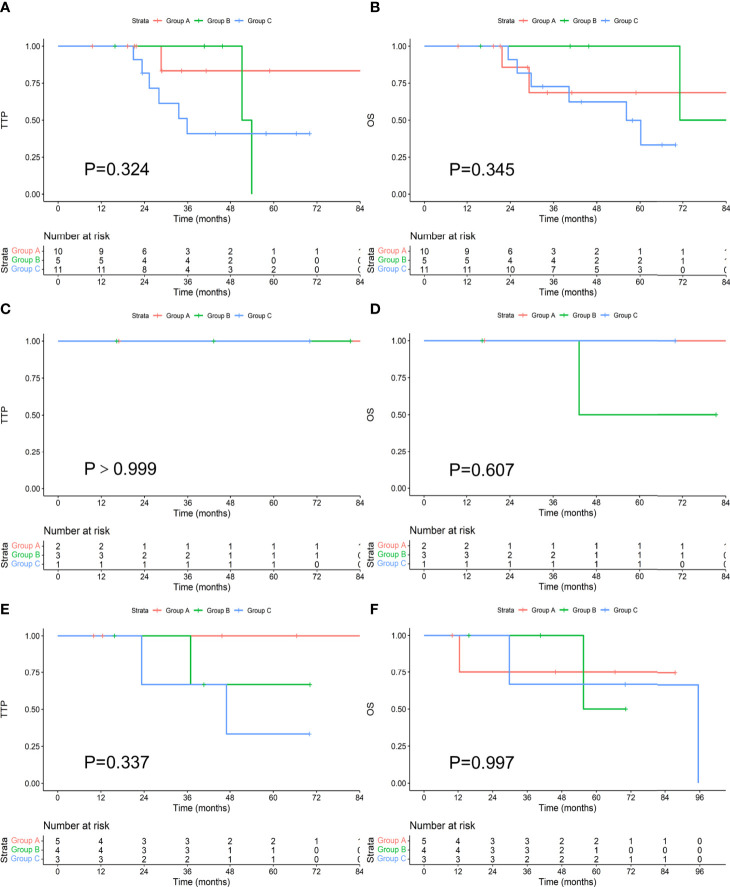
Comparison of TTP **(A, C, E)** and OS **(B, D, F)** in patients with t(4;14), t(14;16), and 17p- who achieved an MRD-negative status at different stages.

There were 26 patients with t(4;14). Among the 10 patients who achieved an MRD-negative status after induction, 1 progressed during the follow-up period, and 2 died. The median TTP and OS were not reached. Among the 5 patients who achieved an MRD-negative status after ASCT, 2 progressed, and 1 died. The median TTP was 52.6 months, and the median OS was 80.1 months. Among the 11 patients who achieved an MRD-negative status after maintenance, 6 progressed, and 6 died. The median TTP was 35.8 months, and the median OS was 56.2 months. There was no difference in TTP or OS between the groups (P values were 0.324 and 0.345, respectively). There were 6 patients with t(14;16), and none of them progressed during the follow-up period. One patient died, and he achieved an MRD-negative status after ASCT. There was no difference in TTP (P>0.999) or OS (P=0.607) between the groups. There were 12 patients with 17p-; among the 5 patients who achieved an MRD-negative status after induction, none progressed, and one died. The median TTP and OS were not reached. Among the 4 patients who achieved an MRD-negative status after ASCT, 1 progressed, and 1 died. The median TTP was not reached, and the median OS was 62.9 months. Of the 3 patients who achieved an MRD-negative status after maintenance, 2 progressed, and 2 died. The median TTP was 47.0 months, and the median OS was 95.5 months. There was no significant difference in TTP or OS between the groups (P values ​​were 0.337 and 0.997, respectively). There was no difference in TTP or OS among those who achieved an MRD-negative status at different stages among the three types of patients with high-risk cytogenetics, as shown in **Figure 5**.

### Survival in Patients With Different R-ISS Stage

**Figure 6:**
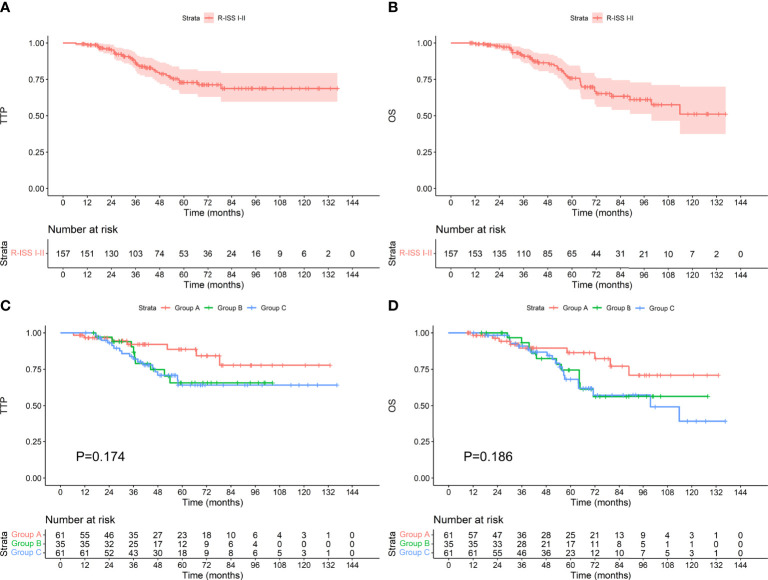
TTP **(A)** and OS **(B)** for patients with R-ISS stage I-II and TTP **(C)** and OS **(D)** for patients who achieved an MRD-negative status at different stages.

**Figure 7:**
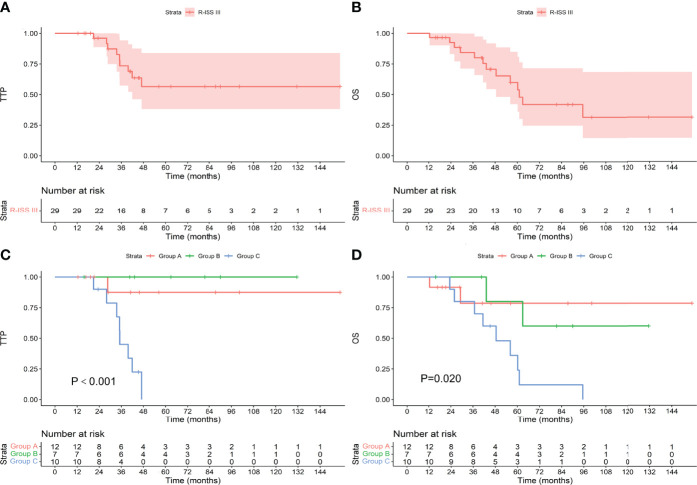
TTP **(A)** and OS **(B)** for patients with R-ISS stage III and TTP **(C)** and OS **(D)** for patients who achieved an MRD-negative status at different stages.

Among the 157 patients with R-ISS stage I-II, the median TTP and OS were not reached. Among the 61 patients who achieved an MRD-negative status after induction, 7 progressed during the follow-up period, and 9 died. The median TTP and OS were not reached. Among the 35 patients who achieved an MRD-negative status after ASCT, 9 progressed, and 11 died. The median TTP and OS were not reached. Among the 61 patients who achieved an MRD-negative status after maintenance, 17 progressed, and 19 died. The median TTP was not reached, and the median OS was 99.6 months. There was no difference in TTP or OS between the groups (as shown in **Figure 6**, P values were 0.174 and 0.186, respectively). Among the 29 patients with R-ISS stage III, the median TTP was not reached, and the median OS was 61.1 months. Among the 12 patients who achieved an MRD-negative status after induction, 1 progressed during the follow-up period, and 2 died. The median TTP and OS were not reached. Among the 7 patients who achieved an MRD-negative status after ASCT, none progressed, and 2 died. The median TTP and OS were not reached. Among the 10 patients who achieved an MRD-negative status after maintenance, 8 progressed, and 9 died. The median TTP was 35.1 months, and the median OS was 48.5 months. As shown in **Figure 7**, there was a significant difference in TTP (Chi-square=15.691, P<0.001) and OS (Chi-square=7.776, P=0.020) between the groups.

### Prognostic Value of The Stage Of Achieving MRD-Negative

COX regression analysis showed that in 186 patients enrolled, high-risk cytogenetics and the stage of reaching MRD-negative were independent prognostic factors for TTP. For patients who achieved MRD-negative after maintenance therapy, compared with those who achieved MRD-negative after induction therapy patients had a shorter TTP. Only age was an independent prognostic factor for OS, but the stage of reaching MRD-negative was not. However, patients who achieved MRD-negative after maintenance therapy had worse OS than those who achieved MRD-negative after induction therapy (**Table 3**, HR=2.409, P=0.025). For patients with R-ISS I-II, there was no prognosis factor for TTP. Age, creatinine, and HBsAg-positive were independent prognostic factors for OS, and the stage of reaching MRD-negative was not a prognostic factor for patients with R-ISS I-II. However, patients who achieved MRD-negative after maintenance therapy had worse OS than those who achieved MRD-negative after induction therapy (**Table 4**, HR=2.289, P=0.043). Univariate analysis showed that the stage of reaching MRD-negative was a risk factor for TTP. There was no prognosis factor for OS. Multivariate analysis showed that the stage of reaching MRD-negative was an independent prognostic factor for OS (P=0.039). Patients who achieved MRD-negative after maintenance therapy had worse OS than those who achieved MRD-negative after induction therapy (**Table 5**, HR=4.922, P=0.044).

**Table 3:** Univariate and multivariate analyses of TTP and OS for enrolled patients.

Covariates	Univariate		Multivariate	
	HR (95% CI)	P	HR (95% CI)	P
TTP				
Age	1.030 (0.993-1.068)	0.112		
Lactate dehydrogenase	0.999 (0.993-1.004)	0.634		
High-risk cytogenetic abnormalities	2.070 (1.007-4.255)	0.048	2.079 (1.011-4.277)	0.047
Bone marrow plasmacytosis	1.007 (0.994-1.021)	0.301		
β2- Microglobulin	1.000 (1.000-1.000)	0.298		
R-ISS III	1.724 (0.824-3.609)	0.148		
Creatinine	0.999 (1.001-1.002)	0.289		
HbsAg positive	1.358 (0.667-2.764)	0.399		
Reinfusion numbers of CD34+ cells	0.947 (0.831-1.080)	0.420		
Induction regiemn	0.871 (0.706-1.076)	0.200		
Maintenance regiemn	0.761 (0.463-1.253)	0.283		
Stage of achieving MRD-negative		0.013		
After induction		0.018		0.011
After ASCT^*^	1.673 (0.645-4.339)	0.289	1.164 (0.402-3.374)	0.779
After maintenance therapy^*^	3.007 (1.354-6.679)	0.007	3.019 (1.332-6.845)	0.008
OS				
Age	1.052 (1.017-1.088)	0.003	1.050 (1.009-1.091)	0.015
Lactate dehydrogenase	1.001 (0.997-1.005)	0.621		
High-risk cytogenetic abnormalities	2.106 (1.078-4.115)	0.029	1.406 (0.697-2.836)	0.342
Bone marrow plasmacytosis	1.007 (0.995-1.019)	0.279		
β2- Microglobulin	1.000048 (1.000050-1.000092)	0.029	1.000 (1.000-1.000)	0.763
R-ISS III	2.062 (1.099-3.867)	0.024	1.321 (0.476-3.669)	0.593
Creatinine	1.999 (1.060-3.768)	0.032	2.014 (0.993-4.083)	0.052
HbsAg positive	1.082 (0.998-3.336)	0.051		
Reinfusion numbers of CD34+ cells	1.036 (0.931-1.153)	0.513		
Induction regimen	1.065 (0.876-1.296)	0.528		
Maintenance regiemn	0.829 (0.507-1.354)	0.454		
Stage of achieving MRD-negative		0.026		
After induction		0.031		0.077
After ASCT^*^	1.666 (0.746-3.721)	0.213	1.604 (0.669-3.843)	0.289
After maintenance therapy^*^	2.523 (1.252-5.084)	0.010	2.409 (1.116-5.201)	0.025

^*^Compared to patients who achieved MRD-negative after induction therapy.

**Table 4:** Univariate and multivariate analyses of TTP and OS for patients with R-ISS I-II.

Covariates	Univariate		Multivariate	
	HR (95% CI)	P	HR (95% CI)	P
TTP				
Age	1.018 (0.979-1.058)	0.367		
Bone marrow plasmacytosis	1.001 (0.984-1.018)	0.897		
Creatinine	1.001 (0.998-1.003)	0.711		
HbsAg positive	1.645 (0.763-3.549)	0.204		
Reinfusion numbers of CD34+ cells	0.958 (0.827-1.110)	0.569		
Induction regiemn	0.902 (0.710-1.146)	0.399		
Maintenance regiemn	0.688 (0.393-1.204)	0.190		
Stage of achieving MRD-negative		0.174		
After induction		0.189		
After ASCT^*^	2.043 (0.760-5.490)	0.157		
After maintenance therapy^*^	2.235 (0.925-5.400)	0.074		
OS				
Age	1.042 (1.004-1.081)	0.029	1.056 (1.018-1.096)	0.004
Bone marrow plasmacytosis	1.004 (0.989-1.019)	0.593		
Creatinine	1.002 (1.000-1.003)	0.100	1.003 (1.001-1.005)	0.010
HbsAg positive	2.721 (1.401-5.287)	0.003	3.275 (1.665-6.443)	0.001
Reinfusion numbers of CD34+ cells	1.044 (0.919-1.186)	0.508		
Induction regimen	1.065 (0.851-1.333)	0.584		
Maintenance regiemn	0.720 (0.403-1.290)	0.270		
Stage of achieving MRD-negative		0.187		
After induction		0.199		0.129
After ASCT^*^	1.829 (0.757-4.417)	0.180	1.667 (0.684-4.061)	0.261
After maintenance therapy^*^	2.051 (0.924-4.450)	0.077	2.289 (1.025-5.108)	0.043

^*^Compared to patients who achieved MRD-negative after induction therapy.

**Table 5:** Univariate and multivariate analyses of TTP and OS for patients with R-ISS III.

Covariates	Univariate		Multivariate	
	HR (95% CI)	P	HR (95% CI)	P
TTP				
Age	1.123 (0.994-1.269)	0.063	1.115 (0.982-1.266)	0.093
Bone marrow plasmacytosis	1.016 (0.989-1.044)	0.237		
Creatinine	1.000 (0.998-1.003)	0.871		
HbsAg positive	0.549 (0.067-4.472)	0.575		
Reinfusion numbers of CD34+ cells	0.950 (0.732-1.234)	0.703		
Induction regiemn	0.803 (0.508-1.269)	0.347		
Maintenance regimen	1.006 (0.377-2.685)	0.991		
Stage of achieving MRD-negative		<0.001		
After induction		0.079		0.072
After ASCT^*^	0.000 (0.000-1.691×10^308)	0.975	0.000 (0.000-8.098×10^302)	0.975
After maintenance therapy^*^	11.325 (1.369-93.665)	0.024	13.330 (1.458-121.848)	0.022
OS				
Age	1.084 (0.990-1.188)	0.083	1.065 (0.970-1.156)	0.197
Bone marrow plasmacytosis	1.005 (0.983-1.026)	0.670		
Creatinine	1.000 (0.998-1.003)	0.837		
HbsAg positive	0.258 (0.032-2.057)	0.201		
Reinfusion numbers of CD34+ cells	1.068 (0.898-1.270)	0.456		
Induction regimen	1.146 (0.732-1.794)	0.550		
Maintenance regimen	1.216 (0.511-2.894)	0.658		
Stage of achieving MRD-negative		0.020		
After induction		0.039		0.039
After ASCT^*^	1.051 (0.147-7.530)	0.961	1.051 (0.147-7.530)	0.961
After maintenance therapy^*^	4.922 (1.045-23.191)	0.044	4.922 (1.045-23.191)	0.044

^*^Compared to patients who achieved MRD-negative after induction therapy.

## Discussion

The detection of MRD has emerged as a significant tool in the management of MM since it has become viewed as highly important for evaluating the response and is strongly associated with PFS and OS (2, 6). However, it is not clear whether there are differences in clinical features and prognosis after reaching an MRD-negative status at different stages.

This study showed that for MM patients who received ASCT, the prognoses of those who achieved an MRD-negative status at different stages were different, and those who achieved an MRD-negative status after induction therapy had a better prognosis than those who achieved MRD-negative after maintenance. In most previous studies, MRD was detected 3 months after ASCT, and the test results were used as the basis for the efficacy evaluation and prognosis prediction (4, 7, 14). However, our research shows that there are differences in the prognoses of patients who achieve an MRD-negative status at different stages. This may be because patients who achieve an MRD-negative status in the initial stage are more sensitive to chemotherapeutics, and subsequent long-term treatment can fully consolidate the therapeutic effect (15). Multivariate analysis showed that in all enrolled patients, the stage of reaching MRD-negative was an independent prognostic factor for TTP, rather than a prognostic factor for OS. In R-ISS I-II patients, the stage of reaching MRD-negative does not predict prognosis. The stage of reaching MRD-negative in patients with R-ISS III was an independent prognostic factor for OS. Although this is not an independent prognostic factor for TTP but the patients who achieved MRD-negative after maintenance therapy had worse TTP. We found that, as in the previous Kaplan-Meier group comparison, patients who achieved MRD-negative after maintenance therapy appeared to have a worse prognosis than those who had MRD-negative after induction therapy.

In patients with high-risk cytogenetics, the stage at which an MRD-negative status is achieved does not affect the prognosis, and there is no difference in patients with standard-risk cytogenetics. In patients with t(4;14), t(14;16), and 17p-, there was no difference in the prognosis of those who achieved an MRD-negative status at different stages. We believe that under the conditions of this study, for patients with high-risk cytogenetics, regardless of the stage at which an MRD-negative is achieved, there is no longer a significant difference in OS. However, it seems that for patients with high-risk cytogenetics, there are certain differences in TTP among those who achieve an MRD-negative status in different stages (P=0.060). This may be because an MRD-negative status is a prognostic factor independent of cytogenetics (14). At present, studies have reported that an MRD-negative status can overcome the prognostic significance of poor cytogenetics, but some studies have reported that an MRD-negative status cannot overcome the prognostic impact of poor cytogenetics (7, 16–19). However, there is currently no research discussing whether the stage at which an MRD-negative status is achieved can predict the prognosis of patients with high-risk cytogenetics; therefore, we believe that our conclusions are worthy of further verification with larger-scale data.

In patients with R-ISS I-II, the stage at which an MRD-negative status is achieved cannot predict the prognosis, but in patients with R-ISS stage III, the stage at which an MRD-negative status is achieved can predict the prognosis. Indeed, achieving undetectable MRD may also abrogate some adverse risk factors, such as R-ISS III (20). Patients who achieve an MRD-negative status after induction therapy have a better prognosis, especially compared with patients who achieve an MRD-negative status after maintenance treatment (21). We found that in high-risk cytogenetics patients, reaching an MRD-negative status cannot predict the prognosis, but it can be in R-ISS III patients. This may be because the R-ISS staging system is a new risk stratification algorithm with improved prognostic power compared with the individual chromosomal abnormality parameters (22). Achievement of an MRD-negative status is affected by both tumor burden and cytogenetics (11, 12). We speculate that for patients with R-ISS III, it is possible to improve long-term survival after ASCT by increasing the MRD negative rate before ASCT by adding induction chemotherapy and other methods (15, 23). We are convinced that the time has come to use it to adapt the treatment strategy to a dynamic risk (10, 20). The current guidelines recommend that patients with NDMM eligible for transplantation require 4–6 courses of induction chemotherapy (1). For patients with R-ISS stage III, it is possible to consider increasing the course of chemotherapy to achieve an MRD-negative status before ASCT to obtain a better prognosis. Although the baseline characteristics and treatment regimens of the patients enrolled in this study were inconsistent, multivariate analysis showed that induction therapy and maintenance therapy were never prognostic factors, so we believe that the conclusions of this study are still of some significance.

In previous studies, MRD results at a single time point were mainly used to predict the prognosis of patients, but an increasing number of studies have found that a single-time MRD test result does not necessarily well predict the prognosis of patients (4, 24). The prediction of prognosis requires multiple cycles and long-term monitoring of MRD. Therefore, at present, some studies are devoted to analyzing the prognostic significance of persistent MRD-negative results, and some studies are focusing on the clinical characteristics of MM patients with persistent MRD-positive results but can survive for a long time (13). Recent research has focused on new methods of MRD detection, such as next-generation flow cytometry, NGS, and liquid biopsy, which have attracted much attention (25–28). These new studies mainly improve the detection depth of MRD by upgrading the detection methods, thereby increasing its clinical value (26). We tested MRD with a popular 10-color flow cytometer and found that even if the stage at which an MRD-negative status was achieved was the same, patients who achieved an MRD-negative status at different stages had different prognostic characteristics. We can predict the prognosis of patients early according to the stage at which an MRD-negative status is achieved.

It should be noted that there were only 29 patients with R-ISS III in this study, which is lower than the ratio of R-ISS III in the population of MM patients because only patients with MRD-negative can be included in this study. After induction therapy-ASCT-maintenance therapy, it is more difficult for patients with R-ISS III to achieve MRD-negative. However, there were statistical differences in the TTP and OS of these 29 patients, the Chi-square was 15.691 and 7.776, respectively, and the 1-β values were both 100%, so the conclusion of the prognostic analysis in R-ISS III patients was established. However, this study was a retrospective clinical study. The clinical characteristics and the process of treatment in included patients were not very consistent, and the number of cases was limited. The conclusions drawn need to be verified in a larger prospective study.

## Data Availability Statement

The original contributions presented in the study are included in the article/supplementary material. Further inquiries can be directed to the corresponding author.

## Ethics Statement

The studies involving human participants were reviewed and approved by Clinical Research and Animal Ethics Committee of The First Affiliated Hospital of Sun Yat-sen University. The patients/participants provided their written informed consent to participate in this study.

## Author Contributions

QS collected the data, analyzed the data, and drafted the manuscript. XL guided the writing and participated in the revision of the manuscript. JG directed the statistical analysis. BH reviews the original data. JRL and MC review and modify the manuscript. JL designed the study, reviewed the research results, and reviewed the manuscript. All authors contributed to the article and approved the submitted version.

## Funding

This study was supported by the National Natural Science Foundation of China (grant number 82070220), Sun Yat-sen University Medical Clinical Trial “5010 Plan” (grant number 2017005), and General Project of the Natural Science Foundation of Guangdong Province (grant number 2021A1515011715).

## Conflict of Interest

The authors declare that the research was conducted in the absence of any commercial or financial relationships that could be construed as a potential conflict of interest.

## Publisher’s Note

All claims expressed in this article are solely those of the authors and do not necessarily represent those of their affiliated organizations, or those of the publisher, the editors and the reviewers. Any product that may be evaluated in this article, or claim that may be made by its manufacturer, is not guaranteed or endorsed by the publisher.
